# Genetic Variation in Coat Colour Genes *MC1R* and *ASIP* Provides Insights Into Domestication and Management of South American Camelids

**DOI:** 10.3389/fgene.2018.00487

**Published:** 2018-11-13

**Authors:** Juan C. Marín, Romina Rivera, Valeria Varas, Jorge Cortés, Ana Agapito, Ana Chero, Alexandra Chávez, Warren E. Johnson, Pablo Orozco-terWengel

**Affiliations:** ^1^Laboratorio de Genómica y Biodiversidad, Departamento de Ciencias Básicas, Universidad del Bío-Bío, Chillán, Chile; ^2^Departamento de Ciencias Básicas, Universidad Santo Tomas, Iquique, Chile; ^3^Doctorado en Ciencias, Mención Ecología y Evolución, Instituto de Ciencias Ambientales & Evolutivas, Facultad de Ciencias, Universidad Austral de Chile, Valdivia, Chile; ^4^Departamento de Zoología, Universidad de Concepción, Concepción, Chile; ^5^Smithsonian Conservation Biology Institute, Smithsonian Institution, Washington, DC, United States; ^6^School of Biosciences, Cardiff University, Cardiff, United Kingdom

**Keywords:** alpaca, llama, vicuña, guanaco, fibre, domestication, hybridization, selection

## Abstract

The domestication of wild vicuña and guanaco by early pre-Inca cultures is an iconic example of wildlife management and domestication in the Americas. Although domestic llamas and alpacas were clearly selected for key, yet distinct, phenotypic traits, the relative patterns and direction of selection and domestication have not been confirmed using genetic approaches. However, the detailed archaeological records from the region suggest that domestication was a process carried out under significant control and planning, which would have facilitated coordinated and thus extremely effective selective pressure to achieve and maintain desired phenotypic traits. Here we link patterns of sequence variation in two well-characterised genes coding for colour variation in vertebrates and interpret the results in the context of domestication in guanacos and vicuñas. We hypothesise that colour variation in wild populations of guanacos and vicunas were strongly selected against. In contrast, variation in coat colour variation in alpaca was strongly selected for and became rapidly fixed in alpacas. In contrast, coat colour variants in llamas were of less economic value, and thus were under less selective pressure. We report for the first time the full sequence of *MC1R* and 3 exons of *ASIP* in 171 wild specimens from throughout their distribution and which represented a range of commonly observed colour patterns. We found a significant difference in the number of non-synonymous substitutions, but not synonymous substitutions among wild and domestics species. The genetic variation in *MC1R* and *ASIP* did not differentiate alpaca from llama due to the high degree of reciprocal introgression, but the combination of 11 substitutions are sufficient to distinguish domestic from wild animals. Although there is gene flow among domestic and wild species, most of the non-synonymous variation in *MC1R* and *ASIP* was not observed in wild species, presumably because these substitutions and the associated colour phenotypes are not effectively transmitted back into wild populations. Therefore, this set of substitutions unequivocally differentiates wild from domestic animals, which will have important practical application in forensic cases involving the poaching of wild vicuñas and guanacos. These markers will also assist in identifying and studying archaeological remains pre- and post-domestication.

## Introduction

The first attempts at domestication coincided with the origins of agriculture some 10,000 years ago. Around that time, a global warming episode marked the end of the last ice age across the planet. More or less simultaneously in several locations of the world, a change from nomadic hunting-gathering to more-sedentary agricultural economies took place, which had a profound impact of human societies and the environment (Gepts and Papa, 2002). Once agricultural societies became more established, their domesticated plants and animals often spread from their original centres of domestication. However, continued contact with wild populations provided ample opportunities for contact and mating with the surrounding wild populations, contributing to the genetic divergence between of the domestic population from its original source population (Currat et al., 2008).

The transition from hunting and gathering to agriculture was a revolutionary inflexion point for humankind, helping support dramatic increases in human population sizes in South America, as in the rest of the world, and facilitating the emergence of modern societies (Larson and Burger, 2013; Goldberg et al., 2016). South America was the last habitable continent colonised by humans and the site of multiple domestication hotspots. Widespread sedentarism began ∼5 ka, coincident with exponential population growth promoted in part by the domestication of potatoes, common beans, peppers, groundnut, cassava, guinea pigs, llamas, and alpacas (Gepts and Papa, 2002). However, compared with the Old World, fewer vertebrate species were domesticated and these were not widely dispersed beyond their original centres of domestication. Arguably the most iconic examples occurred in the Andean high plateau, where the llama (*Lama glama*) was raised primarily as a pack animal and for its fibre and the alpaca (*Vicugna pacos*) was domesticated from the vicuña for its fine fibre.

Camelids (Artiodactyla, Camelidae) currently have both Old and New World representatives. These extant taxa originated from a common ancestor in North America 9–11 million years ago and later radiated into the Nearctic, Neotropical, and Oriental-Ethiopian regions. New World camelids originated from *Hemiauchenia*, from which descendant forms migrated into South America dispersing through either the Llanos of South America or the Cordillera de los Andes. Eventually, the Andes became the major centre for the differentiation of the currently recognised genera *Lama* Cuvier, 1800 (guanaco) and *Vicugna* Lesson, 1842 (vicuña), with guanaco inhabiting the Andes from Peru southward to Patagonia and to the Argentinean Pampa and the Chaco Region and the vicuña the higher elevations of the central Andes (Franklin, 1982; Wheeler, 1991).

*Lama guanicoe*, the largest South American camelid, ranges from sea level to about 3500 m between 8 and 55°S (Franklin, 1982). Two guanaco subspecies are distinguished based on subtle morphological differences. This was confirmed with well-defined genetic differentiation and subspecies designation of populations geographically separated to the northwest (*L. g. cacsilensis*) and southeast (*L. g. guanicoe*) of the central Andes plateau (Marín et al., 2008, 2013). The two vicuña subspecies are distinguished largely by body size, with the northern *V. v. mensalis* being smaller and darker in colour than *V. v. vicugna* (Marín et al., 2007a). The two other species, llama (*L. glama*, Linnaeus 1758) and alpaca (*V. pacos*, Linnaeus 1758), are domestic camelids hypothesised to be derived from *L. guanicoe* and *V. vicugna*, respectively (Kadwell et al., 2001; Wheeler, 2004; Marín et al., 2017).

**FIGURE 1:**
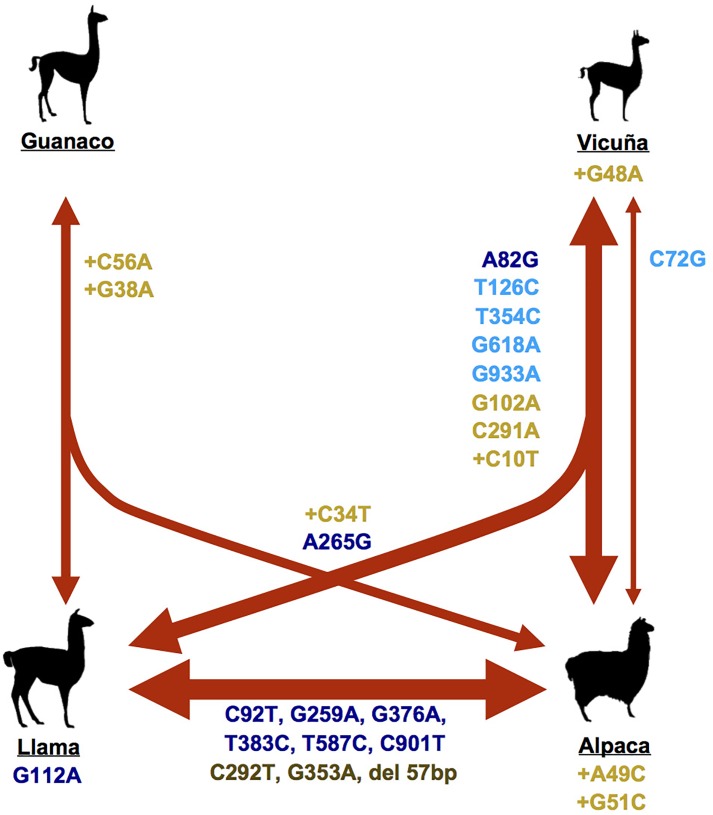
Model of shared and unique substitutions among South American camelids. Names and colours of the polymorphisms correspond to Tables 1, 2. Thickness of the lines are proportional to frequency (Tables 1, 2).

Analysis of incisor morphology from faunal remains from Central Andean archaeological deposits suggests that the vicuña was first brought under human control around 7,000 years ago, leading to the domestic alpaca 1–2,000 years later (Wheeler, 1995). However, other studies have suggested that alpaca descend from guanaco, or that it is a hybrid between llama and vicuña (Hemmer, 1990). In contrast, the ancestry of llama from guanaco has been less controversial. However, it has not been established when and where domestication occurred, in part due to a paucity of archaeological evidence. Because guanaco and llama incisor morphology are identical, osteometric analyses are only able to differentiate small and large camelids among archaeofaunal records (Cartajena et al., 2007; López et al., 2012). At the molecular level, mitochondrial cytochrome b and Control Region sequences do not resolve the phylogenetic relationships among wild and domestic forms of camelids (Stanley et al., 1994; Kadwell et al., 2001; Marín et al., 2007b). In addition, various levels of hybridization among llamas and alpacas have occurred continuously (Kadwell et al., 2001), lessening the genetic distinctions between domestic forms, as well as these with their wild ancestral counterparts (Figure 1).

South American camelid domestication and patterns of genetic diversity were shaped initially by the needs of early Amerindians and later by shifts in breeding practises following the arrival of Europeans and the Spanish conquest (Stanley et al., 1994; Kadwell et al., 2001). It has been widely hypothesised that llamas were first selected for traits associated with producing meat for human consumption and later for their ability to carry heavy loads. In contrast, individual alpacas and their hybrid descendants were likely first selected to improve fibre quality and to produce diverse colours. Although anthropological evidence has provided some insights into these processes and on the timing of domestication, including demographic and selection patterns until now the process of domestication has not been evaluated genetically.

Unlike their wild ancestors, domesticated species are often characterised by a huge allelic variability of coat colour associated genes. This variability occurs largely because artificial selection mitigates the impact of negative pleiotropic effects that otherwise are linked with certain coat colour variants in wild populations (Dong et al., 2015). But artificial selection during domestication is different from a standard selective sweep of a new strongly beneficial variable because artificial selection acts on standing genetic variation, which may have been neutral of even selected against before domestication. Therefore, the fixation of a beneficial allele does not always wipe out DNA variation in the surrounding region and the amount by which variation is reduced largely depends on the initial frequency of the beneficial allele. Recent studies demonstrate that selection for coat colour phenotypes started at the beginning of domestication. Although more than 300 genetic loci and more than 150 coat colour-associated genes have been described, the genetic pathways that determine coat colouration are still poorly understood. However, a few conserved genes have consistently been implicated in coat colour patterns and genes with unique contributions to colour patterns continue to be identified (Cieslak et al., 2011).

Here, we characterised genetic patterns of modern South American camelids in two well-studied coat colour genes, the Melanocortin 1 receptor (*MC1R*) and Agouti-signalling peptide (*ASIP*). Colour patterns of mammalian skin and hair are determined by a relatively small number of genes. These genes have been classified into those active in the development, differentiation, proliferation, and migration of melanocytes and those acting directly on the pigment synthesis. *MC1R* and *ASIP* are two of the best-described genes involved in the synthesis of pigment. *MC1R* encodes the melanocortin receptor that, when coupled to G-proteins, stimulates the production of eumelanin and is responsible for dark colours in melanocytes. *ASIP* produces the agouti signalling protein that acts as an antagonist of *MC1R* by annulling the (∝-MSH) action, thus favouring the production of pheomelanin which produces light colours in melanocytes.

*MC1R* of most vertebrates shares a characteristic allelic hierarchy, where dominant allele (E) produces dark pigment and the recessive allele (e) produces light pigments. Similar mutational patterns have been described in an array of species, including domestic dog (Schmutz et al., 2003), pig (Kijas et al., 1998), and horse (Marklund et al., 1996). In contrast, the dominant *ASIP* allele (A) produces a yellow–red colour pattern while the recessive allele (a) is linked with uniform black. Loss-of-function substitutions in *ASIP* linked with eumelanic phenotypes have been described in several species (Rieder et al., 2001; Eizirik et al., 2003; Kerns et al., 2004; Royo et al., 2008). Although *MC1R* is primarily responsible for determining which pigment type is produced, both *MC1R* and *ASIP* can act locally, causing non-uniform colouration in different regions of the body (Cieslak et al., 2011). Dominant black alleles and putative recessive alleles from the *MC1R* gene have been identified in sheep (Våge et al., 1999; Fontanesi et al., 2011) and goats (Fontanesi et al., 2009). Copy number variation of *ASIP* has also been associated with light and dark coats in goats and sheep (Norris and Whan, 2008; Fontanesi et al., 2011; Dong et al., 2015). In alpaca and llama, several polymorphic sites have been identified on *MC1R* and *ASIP* (Powell et al., 2008; Bathrachalam et al., 2011; Feeley et al., 2011; Daveiro et al., 2016). However, these substitutions have yet to be evaluated in their wild counterparts.

Although guanacos and vicuñas have very homogeneous coat colour patterns, their domestic counterparts exhibit a remarkable range of colours and patterns. However, understanding the process by which these coat colour patterns were selected has been complicated by a lack of molecular markers that discriminate among the four species and by evidence of significant historic gene flow between domestic forms during different time periods. Here we hypothesise that colour variation in wild populations of guanacos and vicunas was strongly selected against. In contrast, variety in coat colour variation in alpaca was strongly selected for, while coat colour variants in llamas were of less economic value thus were under less selective pressure. More specifically, our goal was to identify and quantify patterns of molecular variation in *MC1R* and *ASIP* in the four species of CSA and to determine if there are specific polymorphisms or genetic patterns that are associated with certain colours or that are able to discriminant between the wild and domestic forms of these iconic and economically important South American species. The implications of the results will be discussed in the context of more-recent selection for fibre colour and quality by breeders worldwide.

## Materials and Methods

### Sample Collection and DNA Extraction

Between 1994 and 2016 samples were collected from 82 guanacos and 89 vicuñas from Peru, Bolivia, Argentina, and Chile, encompassing the entire range of distribution of each species (Supplementary Table 1). These samples were complemented with a collection of 89 llamas and 84 alpacas from Ecuador, Peru, Argentina, and Chile were collected and analysed (total dataset 344 samples; Supplementary Table 1). Samples comprised skin (*n* = 4), muscle (*n* = 3), and blood (*n* = 337), and were stored at −70°C in the Laboratorio de Genómica y Biodiversidad, Departamento de Ciencias Básicas, Facultad de Ciencias, Universidad del Bío-Bío, Chillán, Chile or at CONOPA in Lima, Peru. Total genomic DNA was extracted from blood using the Wizard Genomic DNA Purification Kit (Promega, Madison, WI, United States) following the manufacturer’s instructions. DNA from skin and muscle samples was purified using proteinase K digestion and a standard phenol-chloroform protocol (Sambrook et al., 1989). Llamas and alpacas were grouped into six colour groups (black, dark brown, light brown, grey, white, and wild). Sampling permits are provided in the acknowledgements section.

### Amplification and Sequencing of *ASIP* and *MC1R*

Polymerase chain reaction (PCR) primers were designed to amplify the complete *MC1R* coding, following Feeley and Munyard (2009) and the *ASIP* coding region and intronic portions were amplified using primers designed described in Feeley et al. (2011) (Supplementary Table 2). Amplification was performed in a 50 l L reaction volume with ∼30 ng genomic DNA, 1× PCR buffer (8 mM Tris–HCl (pH 8.4); 20 mM KCl (Invitrogen Gibco, Life Technologies), 2 mM MgCl2, 25 l M each of dNTP, 0.5 lM each primer and 0.1U/l Taq polymerase (Invitrogen Gibco, Life Technologies^®^). All PCR amplifications were performed in a Veriti^®^ thermal cycler (Applied Biosystems, Paisley, United Kingdom) with cycling conditions as follows: initial denaturation at 95°C for 10 min, followed by 30–35 cycles of 94°C for 30 s, annealing for 30 s (Supplementary Table 2) and 72°C for 60 s, and a final extension of 72°C for 5 min. PCR products were purified using the GeneClean Turbo for PCR Kit (Bio101) following the manufacturer’s instructions. Products were sequenced in forward and reverse directions using BigDye chemistry on an ABI Prism 3100 semi-automated DNA analyser. The reactions were carried out in a 10 μl volume containing approximately 100 ng of purified DNA, 1 μl of either forward or reverse primer and 2 μl of BigDye Terminator Kit version 3.1 (PerkinElmer). Sequence reactions were visualised using an ABI-3100 sequencer (Perkin Elmer Applied Biosystems).

### Sequence Analysis

Single nucleotide polymorphisms (SNPs) were identified by sequence alignment using Geneious ^1^ and were confirmed by resequencing the whole fragment in the opposite direction. *MC1R* and *ASIP* gene sequences were deposited in GenBank with accession numbers MH596009–MH596352 and MH596353–MH596692). Aligned sequence data for each gene separately were imported into DNASP 5.0 software (Librado and Rozas, 2009) to analyse haplotype diversity and nucleotide diversity. The gametic phase of each haplotype was determined with the software BEAGLE Version 3.3.1 (Browning and Browning, 2007). The genealogical relationship of *MC1R* and *ASIP* haplotypes was described with a haplotype network using the uncorrected median-joining values in Splits Tree4 V 4.14.6 (Huson and Bryant, 2006).

### Detection of Loci Under Selection and Association With Phenotypes

To assess if SNP loci were under selection in the four species of South American camelids, we used BayeScan (Foll and Gaggiotti, 2008), which decomposes locus-population F_ST_ coefficients into a population-specific component (beta) and a locus-specific component (alpha) with positive alpha values indicating diversifying selection. BayeScan was run for each gene with default values under the codominant marker model and assuming two populations, i.e., one with all wild animals and the other with the domestic ones. We also used hapFLK (Fariello et al., 2013) to detect haplotypes under selection, grouping the haplotypes observed in each gene into two populations as done for BayeScan and testing if the haplotype frequencies fit a neutral model. We tested whether the distribution of dN and of synonymous substitutions (dS) in both genes (Tables 1, 2) was the same in the wild and domestic species using chi-square tests calculated for each substitution type separately, and tested if the number of alleles at each polymorphism was the same in the two wild species vs. the two domestic species using Cochran–Mantel–Haenszel tests. Associations between genotypes and coat colour phenotypes were determined using haplo.stats (Sinnwell et al., 2007). Haplo.stats uses an Expectation-Maximisation algorithm to first determine the frequency of haplotypes for each individual and then provides a haplotype-specific score to test for significant differences among cases and controls using a chi-square distribution with degrees of freedom equal to the number of inferred haplotypes in each haplotype block. For these analyses we assessed the correlation of several colour phenotypes in each of the wild and domestic species, however, considering each comparison as a binary system (e.g., animals with black phenotype vs. all animals with a different phenotype). The phenotypes tested were wild (*N* = 171), black (*N* = 15), dark brown (*N* = 15), light brown (*N* = 43), grey (*N* = 4), and white (*N* = 35). Only categories with haplotype frequencies greater than 1% were assessed.

**Table 1:** Single nucleotide polymorphism (SNP) variation detected in *MC1R* of wild and domestics South American camelids.

Polymorphism	Amino acid change	Location	Amino acid effect	Type of substitution	Guanaco	Vicuña	Llama	Alpaca
**C72G**	Leu	c. 72	N/A	**dS**	0 (0)	34 (7)	0 (0)	0 (1)
**A82G**	Thr/Ala	c. 82	Polar to non-polar	**dN**	0 (0)	0 (1)	3 (5)	10 (26)
**C92T**	Thr/Met	c. 92	Polar to non-polar	**dN**	0 (0)	0 (0)	3 (12)	0 (3)
**G112A**	Val/Met	c. 112	Polar to polar	**dN**	0 (0)	0 (0)	0 (13)	0 (0)
**T126C**	Asp	c. 126	N/A	**dS**	0 (0)	2 (2)	2 (6)	9 (29)
**G259A**	Val/Met	c. 259	Non-polar to polar	**dN**	0 (0)	0 (0)	24 (24)	30 (30)
**A265G**	Met/Val	c. 265	Non-polar to polar	**dN**	0 (1)	8 (8)	0 (1)	0 (2)
**T354C**	Asn	c. 354	N/A	**dS**	0 (0)	4 (4)	1 (1)	9 (24)
**G376A**	Gly/Ser	c. 376	Polar to polar	**dN**	0 (0)	0 (0)	19 (24)	27 (30)
**T383C**	Met/Thr	c. 383	Non-polar to polar	**dN**	0 (0)	0 (0)	3 (24)	0 (7)
**T587C**	Phe/Ser	c. 587	Non-polar to polar	**dN**	0 (0)	0 (0)	1 (1)	0 (2)
**G618A**	Leu	c. 618	N/A	**dS**	0 (0)	8 (8)	4 (3)	10 (16)
**C901T**	Arg/Cys	c. 901	Changed to polar	**dN**	0 (0)	3 (0)	5 (0)	14 (8)
**G933A**	Glu	c. 933	N/A	**dS**	13 (0)	70 (3)	7 (2)	24 (1)

*Number of homozygote (heterozygote) individuals for each polymorphic site in guanacos (N = 82), vicuñas (N = 89), llamas (N = 89), and alpacas (N = 84). Type of substitution (dS indicates synonymous substitutions and dN indicates non-synonymous substitutions).*

**Table 2:** Single nucleotide polymorphism variation in *ASIP* of wild and domestic South American camelids.

Polymorphism	Amino acid change	Location	Amino acid effect	Type of substitution	Guanaco	Vicuña	Llama	Alpaca
**G102A**	Gly	c. 102, Exon 2	N/A	**dS**	0 (0)	3 (19)	0 (7)	11 (32)
**+C34T**	N/A	+34, Exon 3	N/A	**dS**	0 (1)	6 (23)	0 (4)	9 (27)
**+G48A**	N/A	+48, Exon 3	N/A	**dS**	0 (0)	0 (15)	0 (0)	0 (0)
**+A49C**	N/A	+49, Exon 3	N/A	**dS**	0 (0)	0 (0)	0 (0)	2 (0)
**+G51C**	N/A	+51, Exon 3	N/A	**dS**	0 (0)	0 (0)	0 (0)	2 (0)
**+C56A**	N/A	+56, Exon 3	N/A	**dS**	28 (21)	0 (1)	38 (16)	29 (19)
**C291A**	Thr	c. 291, Exon 4	N/A	**dS**	0 (0)	12 (26)	1 (3)	11 (25)
**C292T**	Arg/Cys	c. 292, Exon 4	Basic to polar	**dN**	0 (0)	0 (0)	3 (21)	5 (23)
**del 57 bp**	Cys109-Arg127del	325-381, Exon 4	Displacement	**N/A**	0 (0)	0 (0)	23 (17)	6 (24)
**G353A**	Arg/His	c. 353, Exon 4	Basic to polar	**dN**	0 (0)	0 (0)	1 (13)	16 (18)
**+C10T**	N/A	+10, Exon 4	N/A	**dS**	0 (0)	9 (28)	0 (4)	13 (21)
**+G38A**	N/A	+38, Exon 4	N/A	**dS**	30 (17)	0 (1)	40 (9)	21 (25)

*Number of homozygote (heterozygote) individuals for each polymorphism in guanacos (N = 82), vicuñas (N = 89), llamas (N = 89), and alpacas (N = 84). Type of substitution (dS indicates synonymous substitutions and dN indicates non-synonymous substitutions).*

## Results

### MC1R

A fragment of 954bp of the *MC1R* gene was amplified in all samples. Fourteen single nucleotide polymorphisms (SNPs) were identified in this fragment, nine of which presented non-synonymous substitutions (Supplementary Figure 1 and Table 1). Overall, we described 74 haplotypes (Supplementary Table 3) and total haplotype (h) and nucleotide (p) diversity of 0.875 and 0.0037, respectively (Supplementary Table 4). Of the 14 SNPs detected, one (354 T > C) is common in pig (Adeola et al., 2017) and another (SNP 901C > T) has been observed in Old World camelids (Almathen et al., 2018). Substitutions in *MC1R* related with coat colour have also been documented in several domestic mammals (Schmutz et al., 2003; Wang et al., 2013; Adeola et al., 2017; Wu et al., 2017). Our analyses also identified two polymorphisms (c.72 C > G and c.265 A > G) that have not been previously reported in alpacas or llamas but which are present in vicuñas and alpacas. Additionally, one new substitution (c.265 A > G) was detected in low frequencies in the four species (Table 1).

**FIGURE 2:**
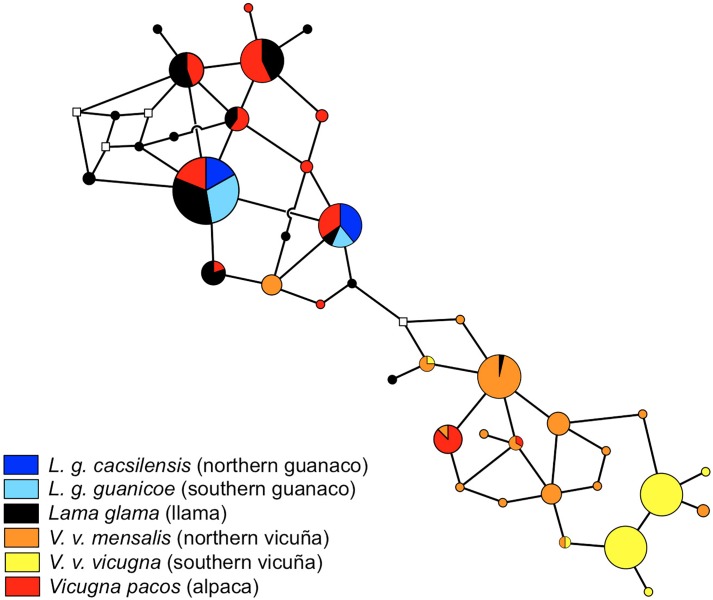
Median-joining network *MC1R* gene genealogy of South American camelids using split decomposition using SplitsTree, version 4.0 (Huson and Bryant, 2006). Circle sizes correspond to haplotype frequencies. The sphere colours correspond to different taxa.

To elucidate the genealogical relationship between the South American Camelids (SAC) *MC1R* haplotypes, we drew a network plot for 39 haplotypes (Figure 2). Overall, a division between the haplotypes in wild *Vicugna* and wild *Lama* species was observed, however, the domestic species shared haplotypes with each other and with their wild relatives. A greater separation between vicuñas from the south and north is evidenced, with the northern ones being closer and sharing more haplotypes with the domestic species, especially alpacas. Moreover, the network did not exhibit a clear genetic partition between llama and alpaca with six haplotypes shared between them, and with more haplotypes shared between the domestic species and *Lama* haplotypes than with *Vicugna* haplotypes.

Translation of the *MC1R* sequence revealed an open reading frame of 318 amino acids. Five of the fourteen SNPs were synonymous substitutions (L24L, D42D, N118N, L206L, and E311E) while the remaining nine resulted in amino acid substitutions (T28A, T31M, V38M, V87M, M89V, G126S, M128T, F196S, and R301C). A comparison between the number of non-synonymous (dN) and synonymous (dS) substitutions among the four species and among the wild and domestics showed an excess of dN substitutions in the domestic species (*p* = 0.0258, and *p* = 0.002, respectively) while no significant differences in the number of dS substitutions (*p* = 0.438, and *p* = 0.4126, respectively). A significant difference in the number of dN and dS among species (*p* = 0.01655) and between the wild and domestics (*p* = 0.0079) was found (Table 3). Of the fourteen substitutions detected in *MC1R*, seven were absent in wild SACs and were found only in domestic species; all of these were non-synonymous changes (Figure 1 and Table 4).

**Table 3:** Number of non-synonymous (dN) and synonymous (dS) substitutions in *MC1R* and *ASIP* among species and among wild and domestic South American camelids.

Species	Number of *MC1R* substitutions		Number of *ASIP* substitutions		Total number of substitutions	
	dN	dS	dN	dS	dN	dS
Wild	4	6	0	10	4	16
Domestic	18	9	6	14	24	23
χ^2^ *p*-values	**0.002838**	0.4386	**0.01431**	0.4142	**0.0001571**	0.2623
Guanaco	1	1	0	3	1	4
Vicuña	3	5	0	7	3	12
Llama	9	4	3	6	12	10
Alpaca	9	5	3	8	12	13
χ^2^ *p*-values dN vs. dS species by species	**0.01656**		**0.002167**		**0.02358**	
χ^2^ *p*-values wild vs. domestic for each genus	**0.00796**		**0.000685**		**0.01087**	
χ^2^ *p*-values wild vs. domestic within substitution type	**0.02588**	0.4126	0.1116	0.5062	**0.002222**	0.1718

*Numbers in bold denote statistically significant values (^∗∗∗^P < 0.05) using χ^2^ test.*

**Table 4:** Polymorphisms with strongest association signals for domestics species.

Species	Non-synonymous substitutions of *MC1R*																		Synonymous substitutions of *MC1R*									
	A82G		C92T		G112A		G259A		A265G		G376A		T383C		T587C		C901T		C72G		T126C		T354C		G618A		G933A	
	A	G	C	T	G	A	G	A	A	G	G	A	T	C	T	C	C	T	C	G	T	C	T	C	G	A	G	A
Guanaco	164	0	82	0	164	0	164	0	163	1	164	0	164	0	164	0	164	0	164	0	0	0	0	0	164	0	0	0
Llama	178	11	160	18	165	13	106	72	177	1	116	62	148	30	175	3	168	10	178	0	168	10	175	3	167	11	164	14
Vicuña	177	75	89	0	178	0	178	0	100	78	178	0	178	0	178	0	172	6	103	75	2	176	4	174	94	84	53	125
Alpaca	122	46	165	3	168	0	75	93	166	2	81	87	161	7	166	2	130	38	167	1	121	47	124	44	129	39	115	53
*p*-value	9.59E-14		0.002738		0.001192		2.58E-47		7.29E-20		3.87E-42		2.32E-09		0.078		5.72E-09		4.13E-20		2.37E-42		4.32E-44		0.000479		5.66E-14	

*Cochran–Mantel–Haenszel association analysis of non-synonymous and synonymous substitutions of MC1R. Association statistics are reported for 14 of the 15 substitutions.*

A significant difference in the number of alleles in the wild vs. domestic species comparison was observed with the Cochran–Mantel–Haenszel test (*p* < 0.001; Table 4) for thirteen of the 14 *MC1R* substitutions. All of the non-synonymous substitutions with non-significant *p*-values occurred at very low frequencies (Table 4). Only 8 of the llamas (8.9%) and 3 of the alpacas (3.6%) had none of the seven substitutions that were unique to the domestic animals. Two of these exclusive domestic substitutions (G259A and G376A) were observed at high frequency (more than 50% of animals) in llamas and alpacas. G259A was present in 52.8% of the llamas and 73.2% of the alpacas; G376A was present in 48.3% of the llamas and 69.5% of the alpacas (Table 4).

Two substitutions (C72G and A265G, synonymous and non-synonymous, respectively) were observed in the southern vicuñas and were very rare in the domestic species (four heterozygous individuals) supporting the hypothesis that alpacas are derived from the northern vicuñas (*V. v. mensalis*). This is further supported by the synonymous substitution G618A which is almost exclusive in northern vicuñas, and is also observed in some alpacas and llamas, but which occurs at very low frequency in the southern vicuñas (Supplementary Table 5).

### ASIP

From the same individuals, we obtained the complete coding sequence of *ASIP* and assessed patterns of variation in a 402-bp gene fragment consisting of 159, 66, and 177 bp of Exons 2, 3, and 4, respectively. In addition, 229-bp of intronic sequence downstream of each exon were also analysed (Supplementary Figure 2 and Table 2). Combined, these constituted 49 haplotypes (Supplementary Table 6) wit total haplotype (h), and nucleotide (p) diversities of 0.782 and 0.004, respectively, for exons and introns combined and nucleotide (p) diversities of 0.0052 and 0.0145 for the exons and introns and haplotypic (h) diversities of 0.433 and 0.709, respectively (Supplementary Table 7).

**FIGURE 3:**
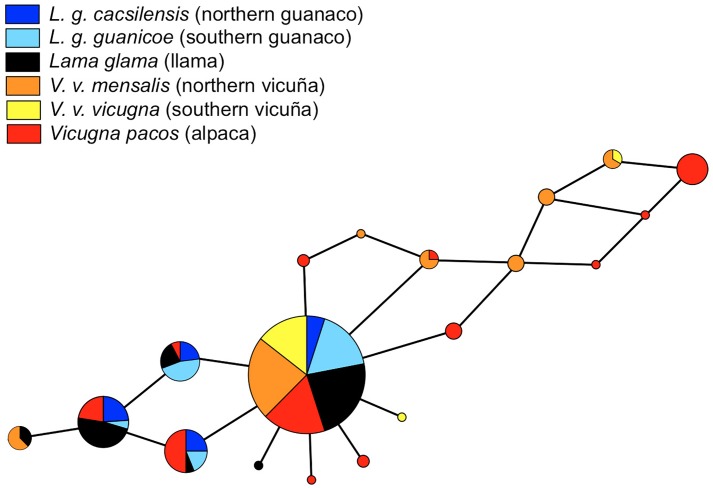
Median-Joining network of *ASIP* gene genealogy of South American camelids using split decomposition using SplitsTree, version 4.0 (Huson and Bryant, 2006). Circle sizes correspond to haplotype frequencies. The sphere colours correspond to different taxa.

The *ASIP* network depicted a relatively simple genealogy with a predominant haplotype shared among all taxa. Unlike the genealogy of *MC1R*, *ASIP* does not show a separation between the *Vicugna* and *Lama* genera, however, ten low frequency and related to each other haplotypes are exclusive to *Vicugna* (Figure 3).

*ASIP* sequences had an open reading frame of 134 amino acids. Two of the 12 substitution cause amino acid changes (C292T and G353A) and one deletion (at position p.C109-Rdel19) is predicted to result in 19 of the 25 amino acids being absent from the mature protein (Table 2). This deletion would likely eliminate the two beta sheets and the R-F-F-motif from the agouti functional domain, which are considered to be essential to interact with ∝-MSH. The other seven observed substitutions are outside the coding region (+C34T, +G48A, +A49C, +G51C, +C56A, C291A, +C10T, and +G38A) or are synonymous changes (G102A).

Nine substitutions were synonymous, one in the Exon 2 (G34G) and one in the Exon 4 (T97T). The others were detected downstream of Exon 3 and 4. Only 2 non-synonymous (dN) substitutions and one deletion of 57 bp were detected in Exon 4. This deletion includes nucleotides 325–381 and would likely mask the polymorphic site G353A (c.325–381 del 57). Additionally, nine synonymous substitutions were detected, seven of which were upstream from exons 3 and 4 and two were in exons 2 and 4 (Supplementary Figure 2). Of the 12 SNPs detected, three have been observed in pig too (Wu et al., 2017). Seven of these substitutions are reported here for the first time in alpacas, llamas, and vicuñas. Only three of these were present in all species and all were synonymous substitutions (Tables 2, 5).

The difference in the number of *ASIP* dN substitutions among wild and domestics was significant (*p* = 0.01431), while that between dS substitutions was not (*p* = 0.4142). A significant difference in the number of dN and dS among species (*p* = 0.00217) and between the wild and domestics (*p* = 0.0007) was found (Table 3). In contrast, both wild and domestic animals present similar levels of dN (*p* = 0.11) and dS (*p* = 0.51) substitutions (Table 3). Of the twelve substitutions detected in *ASIP*, five were absent in the wild, with two of these corresponding to non-synonymous changes (Figure 1 and Table 4).

Significant difference in the allelic count was observed for 7 out 12 substitutions (Cochran–Mantel–Haenszel *p* < 0.001) (Table 5). Five of the substitutions detected in *ASIP* (C292T, G353A, del 57, A+49C, and G+51C) were found only in domestic animals (llamas and/or alpacas). Two of these (C292T and G353A) change the amino acid and the deletion produces a shift in the reading frame. These exclusive domestic substitutions were very common (over 44% of animals), especially in alpacas. Importantly, two dS substitutions of the *ASIP* gene (+C56A and +G38A) discriminated northern (*L. g. cacsilencis*) from southern guanacos (*L. g. guanicoe*). These substitutions were frequently observed in homozygosis in llamas, being observed in 72.7 and 57.1% of llamas and 50.0 and 65.7% of alpacas, respectively, supporting the hypothesis that llamas are derived from the northern guanacos (Supplementary Table 8).

**Table 5:** Polymorphisms with strongest association signals for domestic species.

Species	No-synonymous substitutions of *ASIP*						Synonymous substitutions of *ASIP*																	
	C292T		G353A		del 57 bp		G102A		+C34T		+G48A		+A49C		+G51C		+C56A		C291A		+C10T		+G38A	
	C	T	G	A	+	−	G	A	C	T	G	A	A	C	G	C	C	A	C	A	C	T	G	A
Guanaco	162	0	162	0	162	0	160	0	161	1	160	0	162	0	162	0	85	77	162	0	162	0	85	77
Llama	139	27	161	5	113	63	169	7	172	4	176	0	176	0	176	0	74	102	161	5	162	4	77	89
Vicuña	176	0	176	0	178	0	151	25	141	35	161	15	178	0	176	0	175	1	122	50	126	46	171	1
Alpaca	107	33	90	50	132	36	112	54	123	45	168	0	164	4	164	4	91	77	93	47	93	47	73	67
*p*-value	2.12E-17		2.44E-18		5.42E-26		7.03E-06		0.094		0.000318		0.118		0.12		4.30E-15		0.197		0.104		1.75E-12	

*Cochran–Mantel–Haenszel association analysis of non-synonymous and synonymous substitutions of ASIP. Association statistics are reported for 7 of the 12 substitutions.*

### Detection of Loci Under Selection and Association Analysis

Comparison among wild and domestic species using BayeScan and hapFLK did not identify SNPs under selection. Because of the shared genetic variation due to hybridisation we focused these analyses only on those animals with ≥70 and ≥90% assignment coefficient to their respective group. The BayeScan method didn’t detect outlier loci (FDR = 0.05), however, seven *MC1R* SNPs showed positive alpha values, while only one SNP in *ASIP* showed a positive alpha value. *F_ST_*-values for these SNPs ranged between 0.44 and 0.45 for *M1CR* and between 0.26 and 0.28 for *ASIP*. The hapFLK analysis resulted in *p*-values larger than 0.05 in all tests (Supplementary Figures 3A,B, respectively).

There was a strong association linking *MC1R* haplotypes 1, 2, and 3 and the wild species status (*p* = 3.3e-7, *p* = 5.3e-10, and *p* = 6.7e-12, respectively). Specifically, haplotype 2 was associated with northern and southern guanacos, haplotypes 1 and 3 with northern vicuñas and haplotype 3 was associated with southern vicuñas (*p* = 3.9e-32). Additionally, there was an association of domestic species with haplotypes 4, 5, 6, and 7 (*p* = 7.9e-4, *p* = 1.7e-4, *p* = 1.8e-5, and *p* = 1e-21, respectively). Haplotypes 4 and 5 were linked with alpacas (*p* = 1.2e-10 and *p* = 2.8e-12, respectively) and haplotype 6 with llamas (*p* = 1.7e-10). Haplotype 7 was highly associated with both domestic species (Supplementary Table 9).

With *ASIP* there was an association between haplotype 2 and the white colour in alpacas (*p* = 1.6e-2). Also, haplotype 3 was associated with the variety of light colours (light brown, cream, and white; *p* = 2.3e-6, *p* = 7.3e-4, and *p* = 3.1e-3, respectively). There was no clear association of haplotype 4 with any colour, perhaps because this gene has been mainly linked in other species with dark brown, light brown, cream, and white colours. Other associations were not as robust. However, haplotype 1 was strongly linked with wild species (*p* = 1.9e-40), without discriminating between subspecies. Haplotypes 2, 3, and 4 had a high association with alpaca and llama (*p* = 5.5e-8, *p* = 8.1e-12, and *p* = 9.8e-16, respectively) (Supplementary Table 10).

## Discussion

Archaeological records suggest that the domestication of llamas and alpacas involved very active and directed management practises. This process likely applied strong selection pressure to increase the frequency of desired phenotypic traits, and would have fixed desired (beneficial) phenotypes in the founder populations of domesticated species relatively rapidly. These fixation events differ from the fixation of an advantageous mutant in a natural population, in that artificial selection in a domestication event is likely act on standing genetic variation of the ancestral population which was neutral or nearly neutral before domestication (e.g., Kijas et al., 2012). Contrastingly, through the domestication process it is also possible that selection becomes relaxed as genetic variants under purifying selection in the wild may not be selected against in the protected domestic environment (e.g., Alberto et al., 2018).

South American camelids offer a unique opportunity to study the evolution and domestication of a group of large mammals, particularly since the domestication of llamas and alpacas is relatively well documented and resulted from the selective breeding of two wild species, the guanaco and the vicuña, that still persist. The data generated here supports to a large extent these relationships as shown by our SplitsTree networks for each gene, which reveal similar, yet somewhat contrasting results. Both genes depict the links of llamas with both guanaco subspecies and of the northern vicuna subspecies with the alpaca, however, the resolution differentiating vicuña from guanacos is higher for *MC1R* than *ASIP*. With *ASIP*, the most common haplotype occurs in each of the groups and the majority of the minor haplotypes occurred in alpaca and the northern vicuña. These results are in accordance with the archaeological and cultural records that indicate that llamas were initially bred from guanacos as a source of meat and later for docility and carrying heavy loads and that alpaca were bred from vicuñas for docility and their fine fibre of different colours, becoming essential animals for lifestyle and economy of the Andean people. However, our results demonstrate conclusively that following domestication, llamas and alpacas did not remain genetically isolated and that hybridization between both alpacas and llamas would have occurred regularly, and rare haplotypes, perhaps associated with colour variation, were more-often retained in alpacas.

Overall, we find that the domestic species seem to harbour a substantially higher level of genetic variation than their wild counterparts, and the domestics’ variation has been modified by the effect of artificial selection. In particular, natural selection in the wild is expected to have kept variation in the genes involved in coat colour to a minimum favouring a phenotype of higher fitness, as has been shown for other domestic species (e.g., Gratten et al., 2007; Chessa et al., 2009; Ludwig et al., 2009; Cieslak et al., 2011; Li et al., 2014). However, under domestication, it is expected that variants otherwise removed by selection may have been selected for by humans as phenotypes of interest (Alberto et al., 2018). This is supported by the similarity in the frequency of dN and dS substitutions in a relatively high proportion of SNPs found unique to llamas and alpacas (12 of 26), as well as the genetic variation in wild animals that is also present in domestic animals (Figure 1). It is possible that the substitutions for the coat colour produced in alpacas or vicuñas, particularly those that produce amino acid changes and therefore possibly different phenotypes, were incorporated rapidly into the llamas, possibly with the use of male llamas that were crossed with females alpacas, as previously described with paternal and maternal markers (Marín et al., 2017).

The limited number of polymorphic sites among guanacos across their broad distribution could indicate that there is stronger selection pressure for more-homogeneous colour phenotypes in guanacos, perhaps related with the broad and heterogeneous habitats that guanacos inhabit ranging from the coast (sea-level) to 3,500 m above sea level. In contrast, the large number of shared substitutions among vicuñas and both domestic species (Figure 1) could be an indication that the vicuña underwent strong selection for their fibre quality at a later stage of domestication, eventually being the South American camelids with the smallest diameter of fibre and one of the thinnest fibres among mammals, with fibre diameter of 12.52 ± 1.52 μm (Carpio and Solari, 1982). Similarly, sheep in Eurasia went through at least two successive periods of selection, initially for its meat and 4,000–5,000 years ago for its milk and fine wool (Kijas et al., 2012). Contrary to what is currently common thought, it is possible, that the vicuñas were domesticated after the guanacos when the communities had already satisfied the need for food and became more focus on better clothing. Except for the 57 bp deletion of Exon 4 of the *ASIP* gene, which appears more frequently in llamas, the two substitutions with the strongest signals of selection in *MC1R* gene (G259A and G376A), as well as the other detected substitutions, are more common in alpacas. This pattern that may be a evidence of strong artificial selection (e.g., line breeding) or relaxation of the selection against colour types as fibres of different colours in the alpacas could have generated a great variety of colours that later would have been bred into the llamas as an inevitable consequence of interbreeding.

During the height of the Inca empire, management of domestic and wildlife populations of camelids were likely under relative strict control of central authorities. Agricultural practises were historically maintained by oral tradition and within a specialised group called the *yana*. Animals were segregated and bred based on their desired characteristics and breeding records were recorded with an information storage instrument called the *quipu* (Wheeler et al., 1992). It is not clear to what extent the pre-Columbian alpacas were selected for their fibre diameter or for the purity of colour. However, most of this sophisticated management system and accumulated wisdom would have been lost after the Spanish conquest and as camelids were replaced by traditional sheep breeders, whose main objective was to increase yield (weight) of fibre instead of specific quality or colour. Archaeological remains supporting this scenario have been found in El Yaral, Peru (Wheeler et al., 1995).

The genetic markers and patterns of variation described here have the potential to help identify and protect possible relict populations of pre-conquest alpaca and llama phenotypes (Wheeler et al., 1995). The probable role of hybridization in the evolution of today’s llamas and alpacas is unknown, but it is possible that the 16th century introduction of sheep, cattle, and horses into the region also led to a breakdown in controlled breeding accompanied by extensive hybridization produced by events of the conquest (Wheeler et al., 1995). A study similar to this one, with candidate genes for fibre diameter, will be necessary to test this hypothesis and to be able to develop a full array genetic markers and management tools to assist in improving herds and restoring the local textile industry.

The presence of different coat colours is a typical characteristic of domestic species, it often constitutes one of the phenotypes selected early during domestication in mammals (Fang et al., 2009; Ludwig et al., 2009; Cieslak et al., 2011; Dong et al., 2015), and reflects phenotypes that under wild conditions may be of lower fitness. Consistent with this hypothesis, we find that the number of dN substitutions is significantly lower in wild SACs in comparison with domestic SACs.

Although we know that there is gene flow among the four species, especially between domestic animals or between wild and their domestic derivatives, almost all non-synonymous substitutions were not observed in wild individuals suggesting strong selection against them, or their phenotypes, in guanacos and free-living vicuñas. It is possible then that the selection of special colours in alpacas occurred mainly following domestication, rather than through the capture of albino or melanic vicuñas as has characterised impala, bontebok and wildebeest captive breeding in Southern Africa (Russo et al., 2018).

Notably, the A82G substitution dN of the *MC1R* is present in all the vicuñas and none of the guanacos, clearly differentiating the *Lama* from the *Vicugna*. Two dS substitutions of the same gene (T126C and T354C) show this same distinctive pattern between the two genera. Only some llamas and a greater number of alpacas present the substitution that changes Threonine by Leucine. It is possible that this polymorphism is related to the difference in colour between guanacos and vicuñas, in which the vicuña, with a dark cinnamon colour, could be expressing more pheomelanin than guanacos, whose eumelanin expression could be responsible for the more dark, reddish brown patterns in the southern populations (*L. g. guanicoe*) compared with the lighter brown with ochre yellow tones in the northern variety (*L. g. cacsilensis*) (Wheeler, 2012). The A265G substitution, on the other hand, although it is also scarcely present in guanacos, llamas and alpacas (with 1, 1 and 2 heterozygous individuals, respectively), is a diagnostic substitution for southern vicuñas. This substitution may also be responsible for the tonality differences observed in the southernmost vicuñas, which typically are more yellowish, and which could be related with subtle differences in expression of pheomelanic pigments. Similarly, the dS substitutions C72G and G618A, detected in the *MC1R* gene, together with A265G, are also diagnostic polymorphisms that unequivocally differentiate the two-vicuña subspecies. Interestingly, these three substitutions are heterozygous in individuals inhabiting the contact zone between *V. v. mensalis* and *V. v. vicugna*. Similarly, a less strong signal from the dS + C56A and + G38A substitutions distinguish the more northern from the southernmost guanacos. Since the association between guanaco and llama is not as strong with these substitutions, this may be evidence that guanaco domestication was not primarily motivated by selection for fibre quality or colour.

Finally, although the detected polymorphisms here do not distinguish the domestic species from each other, for the first time, polymorphic sites have been described that distinguish wild South American camelid species from their domestic derivatives. These guanacos and vicuñas haplotypes diagnosed, combined with 10 dN substitutions in llamas and alpacas, will make it possible to distinguish wild from domestic camelid samples. This result will have important implication for forensic applications, including the control of wildlife trade and the conservation of wild species. Application of these techniques and markers will also greatly assist the study of archaeological remains, helping determine the place and time in which the domestication of llamas and alpacas would have occurred. With these results, it will be possible using a panel of 11 substitutions to distinguish a sample of a guanaco or vicuña furtively hunted, or a bone from a llama or alpaca in an archaeological site where its inhabitants have already moved from hunting wild camelids to the breeding of llamas and alpacas. Nevertheless, the absence of a solid association between the substitutions detected here and the different colours present in llamas and alpacas may be due to the existence of the participation of other genes in the expression of this trait. Future studies are needed to combine the analyses done here with similar assessments of polymorphisms in the α-Melanocyte-stimulating hormone (∝-MSH), the Tyrosinase-related protein (TRP1 and 2), the Membrane-Associated Transporter Protein (MATP), and the receptor tyrosine kinases (KIT) in the four species. Genomic studies, on the other hand, will also be able to shed more light on the network of genes involved in this particular phenotype.

## Ethics Statement

Samples were collected following guidelines of the American Society of Mammalogists (Sikes et al., 2011). Specific Permits were required for the Servicio Agrícola y Ganadero, SAG (Permit 447, 2002), the Corporación Nacional Forestal, CONAF (Permit 6/02/2002), for granting other collection permits and helping in collecting samples. The animal research oversight committee of Universidad del Bío-Bío had knowledge of sampling plans prior to their approval of the present animal research protocol. All experimental protocols were approved by the Institutional Animal Care and Use Committee of Universidad del Bío-Bío, the methods were carried out in accordance with the approved guidelines.

## Author Contributions

JM developed the ideas and obtained funding for the project. RR, VV, and JM collected the samples. RR, JC, and AlC conducted the DNA analyses. JM, VV, AA, AnC, WJ, and POTW analysed the data. JM and WJ wrote the paper. All authors read, commented on and approved the final version of the manuscript.

## Conflict of Interest Statement

The authors declare that the research was conducted in the absence of any commercial or financial relationships that could be construed as a potential conflict of interest. The handling Editor is currently co-organising a Research Topic with one of the authors POTW, and confirms the absence of any other collaboration.

## Footnotes

1 www.geneious.com
