# Supplementary material for: Genetic Variation in Coat Colour Genes MC1R and ASIP Provides Insights Into Domestication and Management of South American Camelids
Source: Front Genet. 2018 Nov 13;9:487. doi: 10.3389/fgene.2018.00487 (PMC6242857; doi:10.3389/fgene.2018.00487)

Supplementary Table 1. List of samples of 344 South American camelids analysed. Taxa, localities (ordered approximately from north to south), country and number of individuals.

Taxon	Localities	Country	N = 344
Lama guanicoe cacsilensis			Total = 82
	Huayhua, Departamento de Ayacucho	Perú	7
	Putre, Precordillera de Arica, XV Región	Chile	12
	Ollahue, sector la Chela, II Región	Chile	2
	Zoológico de Chañaral, III Región	Chile	2
	Parque Nacional Llanos de Challe, III Región	Chile	4
	Vallenar, Fundo Socorro (Comuna Alto del Carmen)	Chile	3
	Ovalle, IV Región	Chile	4
	Minera Los Pelambre, Cord. De Illapel, IV Región	Chile	5
	Putaendo, Cord. De San Felipe, V Región	Chile	3
Lama guanicoe guanicoe			
	Uspallata, Provincia de Mendoza	Argentina	3
	Alto Maipo, Región Metropolitana	Chile	3
	La Payunia, Provincia de Mendoza	Argentina	2
	Centro Experimental INIA de Bariloche, Río Negro	Argentina	2
	Meseta Somouncurá, Río Negro	Argentina	2
	Telsen	Argentina	2
	Peninsula Valdés	Argentina	3
	Trelew, sector Bajada del Diablo, Chubut	Argentina	3
	Las Plumas, Chubut	Argentina	1
	Camarones, Chubut	Argentina	1
	Valle Chacabuco, XI Región	Chile	3
	San Julian	Argentina	1
	Parque Nacional Torres del Paine, XII Región	Chile	5
	Islas Falkland	Argentina	1
	Sector Rufin, Tierra del Fuego, XII Región	Chile	3
	Sector Calafate, Tierra del Fuego, XII Región	Chile	2
	Porvenir, Tierra del Fuego, XII Región	Chile	3
Lama glama			Total = 89
	INTA Abrapampa, Provincia de Jujuy	Argentina	1
	Marangani, Departamento de Cuzco	Perú	3
	Marcapomacocha, Departamento de Junín	Perú	5
	Chimborazo, Provincia de Chimborazo	Ecuador	2
	Llamas del Sur, IX Región	Chile	78
Vicugna vicugna mensalis			Total = 89
	Catac, Departamento de Ancash	Perú	5
	Sto. Domingo de Cachi Cachi, Departamento de Junín	Perú	6
	Cerro Azul, Departamento de Cuzco	Perú	4
	UNH, Departamento de Huancavelica	Perú	3
	San Pedro de Huarcarpana, Departamento de Ica	Perú	2
	Reserva Nacional de Pampa Galera, Depto de Ayacucho	Perú	5
	Jauja, Cachi Cachi, Departamento de Junín	Perú	2
	Ayavi-Tambo-Huaytará, Depto Huancavelica	Perú	5
	Ingenio, Huacullani, Departamento de Puno	Perú	3
	S.A.I.S. Picotani, Departamento de Puno	Perú	2
	Tinco Cancha, Yauli, Departamento de Junín	Perú	3
	Corral Ankara(Pakiza), XV Región	Chile	3
	Parque Nacional Lauca, XV Región	Chile	6
	Lagunillas, XV Región	Chile	4
	Salar de Surire, XV Región	Chile	4
Vicugna vicugna vicugna			
	Santa Catalina, Provincia de Jujuy	Argentina	2
	Cieneguillas, Provincia de Jujuy	Argentina	7
	INTA Abrapampa, Provincia de Jujuy	Argentina	4
	Parque Nacional Llulaillaco, II Región	Chile	4
	Laguna Blanca, Provincia de Catamarca	Argentina	5
	Corral Ankara, XV Región	Chile	2
	Parque Nacional Nevado Tres Cruces, III Región	Chile	3
	Reserva Prov. San Guillermo, Prov. de San Juan	Argentina	5
Vicugna pacos			Total = 84
	Pisacoma	Perú	1
	Santa Rosa de Melgar	Perú	1
	Patambuco	Perú	1
	Pachacayo, Junín	Perú	8
	"M. Duran" Zoo	Chile	2
	Breeding farm Puchi	Chile	5
	Breeding farm "Llamas del Sur", IX Región	Chile	66

Supplementary Table 2. List of primers used for polymerase chain reaction (PCR) and sequencing.

Primer name	Sequence (5´ to 3´)	Tm (ºC)	Size (bp)	Referencia
**MC1R**				
MC1R3-F	GGGAGAAGGTGAGTGTGAGG	65	700	Feeley 2009
MC1R-R	GTGGACCGCTACATCTCCAT			
MC1R-F	AGAGGAGGACGGCTGTGTGA	60	750	Feeley 2009
MC1R4-R	GCTCTTCCTGGAGATTCGTG			
**ASIP**				
Ex2-F	CTCAACTGGGACACTTGTGG	60	416	Feeley 2011
Ex2-R	AGCACAAAGGAGCTGTGACC			
Ex3-F	TCTATTCAGCCAACCCTTCG	60	350	Feeley 2011
Ex3-R	GGTCTGGTCAGAGCTCAAGG			
Ex4-F	TAAGTCCGAGCAGGTAGTGG	65	560	Feeley 2011
Ex4-R	AGGGAGCATGTGCGTAGC			

Supplementary Table 3. Alignment of 76 haplotypes showing substitutions of *MC1R* gene. Frequency for each haplotype in guanacos (g, N=82), vicuñas (v, N=89), llamas (l, N=89) and alpacas (a, N=84).

Haplotype	C72G	A82G	C92T	G112A	T126C	G259A	A265G	T354C	G376A	T383C	T587C	G618A	C901T	G933A	Specie(s)	Frequency
1	C	G	C	G	T	G	A	C	G	T	T	G	C	G	v	0,00145
2	C	G	C	G	C	G	G	C	G	T	T	A	C	A	v	0,00145
3	C	G	C	G	T	G	A	C	G	T	T	A	C	G	v	0,00145
4	G	G	C	G	C	G	A	C	G	T	T	G	C	A	v	0,00145
5	C	A	C	G	C	G	A	C	G	T	T	A	C	A	v	0,00145
6	G	G	C	G	C	G	G	T	G	T	T	G	C	G	v	0,00145
7	C	G	C	G	C	G	A	T	G	T	T	A	C	G	l	0,00145
8	C	G	C	G	C	G	A	T	G	T	C	A	C	G	l	0,00145
9	C	A	C	G	T	A	A	T	A	C	T	G	C	A	l	0,00145
10	C	A	C	G	T	A	A	T	G	T	T	G	T	A	l	0,00145
11	C	A	T	G	T	A	A	T	G	T	T	G	C	G	l	0,00145
12	C	A	C	G	T	A	A	T	G	T	T	A	C	G	l	0,00145
13	C	A	T	G	T	A	A	T	G	C	T	A	C	G	l	0,00145
14	C	A	T	G	T	G	A	T	G	T	T	G	T	G	l	0,00145
15	C	A	T	G	T	G	A	T	G	C	T	A	C	A	l	0,00145
16	C	A	C	G	T	G	A	T	G	T	T	A	C	A	l	0,00145
17	C	A	C	G	T	G	A	T	G	C	T	G	C	A	l	0,00145
18	C	A	C	A	T	G	A	T	G	C	T	G	C	G	l	0,00145
19	C	A	T	G	C	G	A	T	G	T	T	G	T	G	l	0,00145
20	C	A	C	G	C	A	A	T	A	T	T	A	C	A	a	0,00145
21	C	G	C	G	C	A	A	T	G	T	T	G	C	G	a	0,00145
22	C	A	C	G	T	G	G	T	G	T	T	G	C	A	a	0,00145
23	C	G	C	G	C	G	A	C	G	T	T	G	T	A	a	0,00145
24	C	A	C	G	T	A	G	T	G	T	T	G	C	A	a	0,00145
25	C	A	C	G	T	A	A	T	A	C	T	G	T	A	a	0,00145
26	C	A	C	G	T	A	A	T	A	C	C	G	C	G	a	0,00145
27	G	A	C	G	C	A	A	T	G	T	T	G	C	G	a	0,00145
28	C	A	C	G	C	A	A	T	A	T	T	G	C	G	a	0,00145
29	C	G	C	G	C	A	A	C	G	T	T	A	T	A	a	0,00145
30	C	G	C	G	T	G	A	C	A	T	T	G	C	G	a	0,00145
31	C	A	C	G	T	G	A	T	G	T	T	A	T	A	a	0,00145
32	C	A	C	G	C	G	A	T	G	T	T	G	C	G	a	0,00145
33	C	A	C	G	T	G	A	T	G	C	T	G	T	A	a	0,00145
34	C	A	C	A	T	G	A	T	G	T	T	G	C	G	l	0,01453
35	C	A	C	G	C	G	A	C	G	T	T	A	T	A	a	0,00291
36	C	A	C	G	T	A	A	T	A	C	T	G	C	G	l-a	0,00581
37	C	A	C	G	T	A	A	T	A	T	T	G	C	A	l-a	0,01599

38	C	A	C	G	T	A	A	T	A	T	T	G	C	G	l-a	0,15116
39	C	A	C	G	T	A	A	T	A	T	T	G	T	A	l-a	0,00581
40	C	A	C	G	T	A	A	T	A	T	T	G	T	G	l-a	0,00727
41	C	A	C	G	T	A	A	T	G	C	T	G	C	G	l	0,00291
42	C	A	C	G	T	A	A	T	G	T	T	G	C	A	l-a	0,00291
43	C	A	C	G	T	A	A	T	G	T	T	G	C	G	l-a	0,01744
44	C	A	C	G	T	G	A	C	G	T	T	G	C	G	a	0,00291
45	C	A	C	G	T	G	A	T	A	C	T	G	C	G	l	0,00291
46	C	A	C	G	T	G	A	T	A	T	T	G	C	G	l-a	0,01017
47	C	A	C	G	T	G	A	T	G	C	T	G	C	G	l-a	0,02035
48	C	A	C	G	T	G	A	T	G	C	T	G	T	G	l	0,00436
49	C	A	C	G	T	G	A	T	G	T	T	G	C	A	g-l-a	0,04070
50	C	A	C	G	T	G	A	T	G	T	T	G	C	G	g-l-a	0,57267
51	C	A	C	G	T	G	A	T	G	T	T	G	T	A	a	0,00291
52	C	A	C	G	T	G	A	T	G	T	T	G	T	G	l-a	0,00436
53	C	A	C	G	T	G	G	T	G	T	T	G	C	G	g-l	0,00291
54	C	A	T	A	T	G	A	T	G	T	T	G	C	G	l	0,00291
55	C	A	T	G	T	A	A	T	G	C	T	G	C	G	l	0,00291
56	C	A	T	G	T	G	A	T	G	C	T	G	C	G	l-a	0,00291
57	C	A	T	G	T	G	A	T	G	T	T	G	C	G	l-a	0,01453
58	C	G	C	G	C	G	A	C	G	T	T	A	C	A	v-l-a	0,10610
59	C	G	C	G	C	G	A	C	G	T	T	A	C	G	v-a	0,01599
60	C	G	C	G	C	G	A	C	G	T	T	A	T	A	v-a	0,02616
61	C	G	C	G	C	G	A	C	G	T	T	A	T	G	v-a	0,00581
62	C	G	C	G	C	G	A	C	G	T	T	G	C	A	v	0,00727
63	C	G	C	G	C	G	A	C	G	T	T	G	C	G	v-a	0,02471
64	C	G	C	G	C	G	A	C	G	T	T	G	T	G	v-a	0,00436
65	C	G	C	G	C	G	A	T	G	T	C	A	C	A	l-a	0,00436
66	C	G	C	G	C	G	A	T	G	T	T	A	C	A	v	0,00436
67	C	G	C	G	C	G	A	T	G	T	T	G	C	G	l-a	0,00581
68	C	G	C	G	C	G	G	C	G	T	T	G	C	G	v	0,00581
69	C	G	C	G	T	A	A	T	A	T	T	G	C	G	l-a	0,00436
70	C	G	C	G	T	G	A	T	G	T	T	G	C	G	a	0,00436
71	G	G	C	G	C	G	G	C	G	T	T	A	C	A	v	0,00436
72	G	G	C	G	C	G	G	C	G	T	T	G	C	A	v-l	0,06686
73	G	G	C	G	C	G	G	C	G	T	T	G	C	G	v	0,03343
74	G	G	C	G	C	G	G	C	G	T	T	A	C	G	v	0,00291

Supplementary Table 4. Genetic diversity indices of 344 South American camelids in *MC1R* gene. n, number of samples; na, number of haplotypes observed; np, number of private haplotypes; h, haplotype diversity; Π, nucleotide diversity; p, number of polymorphic sites; pp, private polymorphic; Tajima's *D*; Fu's *Fs*.

Species or subspecies	n	na	np	h ± (SD)	Π ± (SD)	p	Tajima's *D*	Fu's *Fs*
Guanaco	82	3	0	0.279 ± (0.04)	0.00029 ± (0.00004)	2	-0.28721 p>0.10	-1.05850 p>0.10
Northern guanaco	23	2	0	0.464 ± (0.045)	0.00049± (0.00203)	1	1.42600 p>0.10	0.55053 p>0.10
Southern guanaco	59	3	0	0.169 ± (0.044)	0.00018± (0.00005)	1	-0.80626 p>0.10	-1.11338 p>0.10
Vicuña	89	20	0	0.794 ± (0.020)	0.00217 ± (0.00005)	9	0.75401 p>0.10	0.67886 p>0.10
Northern vicuña	57	17	0	0.667 ± (0.048)	0.00146± (0.00014)	8	-0.17813 p>0.10	0.42779 p>0.10
Southern vicuña	32	6	0	0.578 ± (0.040)	0.00071± (0.0008)	5	-0.82092 p>0.10	-0.92643 p>0.10
Llama	89	34	13	0.852 ± (0.017)	0.00232 ± (0.00014)	13	-0.05154 p>0.10	0.60284 p>0.10
Alpaca	84	34	2	0.858 ± (0.022)	0.00363 ± (0.00017)	13	1.30265 p>0.10	1.21163 p>0.10
Wild species	171	23	0	0.779 ± (0.017)	0.00327 ± (0.00006)	9	2.61112 p<0.05	2.08784 p<0.02
Domestics species	173	52	22	0.865 ± (0.014)	0.00311 ± (0.00013)	14	0.85227 p>0.10	0.99764 p>0.10
TOTAL	344	66	-	0.875 ± (0.008)	0.00370 ± (0.00008)	15	1.52815 p>0.10	1.83175 p<0.05

Supplementary Table 5. Allele frequencies within each group of the 14 SNPs of synonymous and no synonymous *MC1R* gene detected in wild and domestic South American camelids by species and subspecies. Standard deviation including all individuals in parentheses.

**Specie or subspecie**	**C72G**		**A82G**		**C92T**		**G112A**		**T126C**		**G259A**		**A265G**		**T354C**		**G376A**		**T383C**		**T587C**		**G618A**		**C901T**		**G933A**	
	C	G	A	G	C	T	G	A	T	C	G	A	A	G	T	C	G	A	T	C	T	C	G	A	C	T	G	A
Guanaco	1	0	1	0	1	0	1	0	1	0	1	0	0.99	0.01	1	0	1	0	1	0	1	0	1	0	1	0	0.84	0.15
Northern guanaco	1	0	1	0	1	0	1	0	1	0	1	0	1	0	1	0	1	0	1	0	1	0	1	0	1	0	0.65	0.35
Southern guanaco	1	0	1	0	1	0	1	0	1	0	1	0	0.99	0.01	1	0	1	0	1	0	1	0	1	0	1	0	0.92	0.08
Vicuña	0.57	0.42	0.99	0.05	1	0	1	0	0.01	0.98	1	0	0.56	0.43	0.02	0.97	1	0	1	0	1	0	0.52	0.47	0.96	0.03	0.29	0.70
Northern vicuña	0.89	0.11	0.01	0.99	1	0	1	0	0.02	0.98	1	0	0.88	0.12	0.03	0.97	1	0	1	0	1	0	0.17	0.83	0.95	0.05	0.09	0.91
Southern vicuña	0.03	0.97	0	1	1	0	1	0	0	1	1	0	0	1	0.02	0.98	1	0	1	0	1	0	0.98	0.02	1	0	0.39	0.61
Llama	1	0	0.93	0.06	0.89	0.10	0.92	0.07	0.94	0.05	0.59	0.40	0.99	0.005	0.98	0.01	0.65	0.34	0.83	0.16	0.98	0.01	0.93	0.06	0.94	0.05	0.92	0.07
Alpaca	0.99	0.005	0.73	0.26	0.98	0.01	1	0	0.72	0.27	0.46	0.53	0.98	0.01	0.75	0.24	0.49	0.50	0.95	0.04	0.98	0.01	0.78	0.21	0.78	0.21	0.70	0.29
Total	0.89 (0.21)	0.10 (0.21)	0.91 (0.12)	0.083 (0.12)	0.97 (0.04)	0.02 (0.04)	0.98 (0.03)	0.01 (0.03)	0.66 (0.45)	0.33 (0.45)	0.76 (0.27)	0.23 (0.27)	0.88 (0.21)	0.11 (0.21)	0.69 (0.45)	0.30 (0.45)	0.78 (0.25)	0.21 (0.25)	0.94 (0.07)	0.05 (0.07)	0.99 (0.008)	0.007 (0.009)	0.81 (0.21)	0.18 (0.21)	0.92 (0.09)	0.07 (0.09)	0.69 (0.27)	0.30 (0.27)

Supplementary Table 6. Alignment of 76 haplotypes showing substitutions of *ASIP* gene. Frequency for each haplotype in guanacos (g, N=82), vicuñas (v, N=89), llamas (l, N=89) and alpacas (a, N=84).

Haplotype	**G102A**	**+C34T**	**+G48A**	**+A49C**	**+G51C**	**+C56A**	**C291A**	**C292T**	**G353A**	**del57bp**	**+C10T**	**+G38A**	Specie(s)	Frequency
1	G	T	G	A	G	C	C	C	G	+	C	G	g	0,00146
2	G	C	A	A	G	C	G	C	G	+	T	G	v	0,00146
3	G	C	G	A	G	C	C	C	G	+	T	G	v	0,00146
4	G	C	A	A	G	C	G	C	G	+	C	G	v	0,00146
5	A	T	G	A	G	C	C	C	A	+	T	A	l	0,00146
6	G	T	G	A	G	A	C	C	G	+	C	A	l	0,00146
7	A	C	G	A	G	C	C	C	G	+	C	A	l	0,00146
8	A	C	G	A	G	A	C	T	G	+	C	A	a	0,00146
9	G	C	G	A	G	C	C	C	G	+	T	A	a	0,00146
10	G	C	G	A	G	C	C	T	G	-	C	G	a	0,00146
11	G	C	G	A	G	A	G	C	A	+	T	G	a	0,00146
12	G	C	G	A	G	C	G	C	A	+	T	G	a	0,00146
13	A	T	G	A	G	C	G	T	G	+	C	A	a	0,00146
14	A	C	G	A	G	C	G	T	G	+	C	A	a	0,00146
15	G	T	G	A	G	C	G	C	A	+	T	G	a	0,00146
16	A	C	G	A	G	C	G	C	A	+	C	G	a	0,00146
17	A	C	G	A	G	C	C	T	G	+	C	A	a	0,00146
18	A	C	G	A	G	A	C	C	G	+	C	G	a	0,00146
19	A	T	G	A	G	C	G	C	A	+	T	A	a	0,00146
20	A	C	G	C	C	C	C	C	A	+	T	G	a	0,00146
21	G	C	G	C	C	C	G	C	A	-	C	G	a	0,00146
22	A	C	G	A	G	C	C	C	G	+	C	G	a	0,00292
23	A	C	G	A	G	C	C	C	G	-	C	G	a-l	0,00439
24	A	C	G	A	G	C	G	C	A	+	T	G	a-l	0,00439
25	A	T	A	A	G	C	G	C	G	+	T	G	v	0,00731
26	A	T	G	A	G	A	G	C	A	+	T	G	a	0,01023
27	A	T	G	A	G	A	G	C	G	+	T	G	v-a	0,00292
28	A	T	G	A	G	C	G	C	A	+	T	G	l-a	0,04678
29	A	T	G	A	G	C	G	C	A	-	T	G	a	0,00292
30	A	T	G	A	G	C	G	C	G	+	T	G	v-l-a	0,03070
31	G	C	A	A	G	C	C	C	G	+	C	G	v	0,01170
32	G	C	G	A	G	A	C	C	G	+	C	A	g-a-l	0,23830
33	G	C	G	A	G	A	C	C	G	+	C	G	g-a-l	0,03509

34	G	C	G	A	G	A	C	C	G	-	C	G	l	0,00877
35	G	C	G	A	G	A	C	T	G	+	C	A	a-l	0,06871
36	G	C	G	A	G	A	C	T	G	+	C	G	a-l	0,00292
37	G	C	G	A	G	A	C	T	G	-	C	G	a-l	0,00292
38	G	C	G	A	G	C	C	C	A	+	C	G	a-l	0,00439
39	G	C	G	A	G	C	C	C	A	-	C	G	a	0,00439
40	G	C	G	A	G	C	C	C	A	-	T	A	a	0,00292
41	G	C	G	A	G	C	C	C	G	+	C	A	g-v-l-a	0,02778
42	G	C	G	A	G	C	C	C	G	+	C	G	g-v-l-a	0,29094
43	G	C	G	A	G	C	C	C	G	-	C	A	l	0,00292
44	G	C	G	A	G	C	C	C	G	-	C	G	a-l	0,10819
45	G	C	G	A	G	C	C	T	G	+	C	A	a	0,00585
46	G	C	G	A	G	C	G	C	G	+	C	G	v	0,00585
47	G	C	G	A	G	C	G	C	G	+	T	G	v-a	0,01462
48	G	C	G	A	G	C	G	C	G	-	C	G	l	0,00292
49	G	C	G	C	C	C	C	C	G	+	C	G	a	0,00292

Supplementary Table 7. Genetic diversity indices of 342 South American camelids in *ASIP*. n, number of samples; na, number of haplotypes observed; np, number of private haplotypes; h, total haplotype diversity; Π, total nucleotide diversity; p, number of polymorphic sites; pp private polymorphic; Tajima's *D*; Fu's *Fs*. Haplotype and nucleotide diversity of exons and introns in shown under the superindices E and I respectively.

Specie or subspecies	n	na	np	h ± (SD)	Π	$h \pm (SD)^E$	$h \pm (SD)^I$	$\Pi \pm (SD)^E$	$\Pi \pm (SD)^I$	p	Tajima's *D*	Fu's *Fs*
Guanaco	81	5	0	0.647 ± (0.02)	0.00201 ± (0.0003)	0.00 ± (0.00)	0.647 ± (0.02)	0.00 ± (0.00)	0.00987 ± (0.00015)	3	1.51995 p>0.10	0.02712 p>0.10
Northern guanaco	22	4	0	0.447 ±(0.080)	0.00123 ± (0.00085)	0.00 ± (0.00)	0.443 ± (0.08)	0.00 ± (0.00)	0.00602± (0.00136)	3	-0.21350 p>0.10	-0.37752 p>0.10
Southern guanaco	59	4	0	0.593 ±(0.035)	0.00181 ±(0.0001)	0.00 ± (0.00)	0.593 ± (0.035)	0.00 ± (0.00)	0.00887± (0.00047)	2	2.14986 p<0.05	0.67279 p>0.10
Vicuña	88	14	0	0.591 ±(0.042)	0.00320 ±(0.00139)	0.451 ± (0.040)	0.536 ± (0.041)	0.00161 ± (0.00015)	0.00939 ± (0.00085)	11	-0.37728 p>0.10	-1.49949 p>0.10
Northern vicuña	56	11	0	0.689 ±(0.042)	0.00368 ±(0.0028)	0.555 ± (0.040)	0.614 ± (0.043)	0.00197± (0.00016)	0.01034± (0.00086)	7	0.92939 p>0.10	-0.59045 p>0.10
Southern vicuña	32	8	0	0.363 ±(0.076)	0.00196 ±(0.00050)	0.204 ± (0.065)	0.359 ± (0.074)	0.00072± (0.00025)	0.00681± (0.00165)	9	-1.28548 p>0.10	-0.04604 p>0.10
Llama	89	16	0	0.740 ± (0.019)	0.00302 ±(0.00112)	0.354 ± (0.042)	0.0606 ± (0.022)	0.00359 ± (0.00245)	0.01050 ± (0.00044)	8	0.21978 p>0.10	1.21777 p>0.10
Alpaca	84	27	1	0.880 ± (0.012)	0.00712 ±(0.00144)	0.698 ± (0.028)	0.783 ± (0.020)	0.01438 ± (0.00491)	0.01870 ± (0.00065)	12	-0.37728 p>0.10	-1.44949 p>0.10
Wild species	169	17	0	0.673 ± (0.024)	0.00328 ±(0.00121)	0.264 ± (0.030)	0.651 ± (0.025)	0.00096 ± (0.00012)	0.01231 ± (0.00066)	11	-0.08464 p>0.10	-0.21548 p>0.10
Domestics species	173	30	0	0.828 ± (0.012)	0.00539 ±(0.00127)	0.557 ± (0.029)	0.707 ± (0.017)	0.00914 ± (0.00274)	0.01524 ± (0.00055)	12	1.04572 p>0.10	0.65168 p>0.10
TOTAL	342	38	-	0.782 ± (0.0012)	0.00455 ±(0.00141)	0.433 ± (0.023)	0.709 ± (0.012)	0.00520 ± (0.0000)	0.01450 ± (0.0000)	17	-0.09074 p>0.10	-0.39629 p<0.10

Supplementary Table 8. Allele frequencies within each group of 12 SNPs of synonymous and no synonymous *ASIP* gene detected in wild and domestics South American camelids by species and subspecies. Standard deviation including all individuals in parentheses.

**Specie or subspecie**	**G102A**		**+C34T**		**+G48A**		**+A49C**		**+G51C**		**+C56A**		**C291A**		**C292T**		**G353A**		**del 57bp**		**+C10T**		**+G38A**	
	G	A	C	T	G	A	A	C	G	C	C	A	C	A	C	T	G	A	+	-	C	T	G	A
Guanaco	1	0	0.99	0.006	1	0	1	0	1	0	0.52	0.47	1	0	1	0	1	0	1	0	1	0	0.52	0.47
Northern guanaco	1	0	0.98	0.02	1	0	1	0	1	0	0.91	0.09	1	0	1	0	1	0	1	0	1	0	0.27	0.73
Southern guanaco	1	0	1	0	1	0	1	0	1	0	0.69	0.31	1	0	1	0	1	0	1	0	1	0	0.62	0.38
Vicuña	0.85	0.14	0.80	0.19	0.91	0.08	1	0	1	0	0.99	0.005	0.70	0.29	1	0	1	0	1	0	0.73	0.26	0.99	0.005
Northern vicuña	0.80	0.20	0.75	0.25	0.89	0.11	1	0	1	0	0.99	0.01	0.61	0.39	1	0	1	0	1	0	0.65	0.35	0.99	0.01
Southern vicuña	0.95	0.05	0.89	0.11	0.95	0.05	1	0	1	0	1	0	0.89	0.11	1	0	1	0	1	0	0.89	0.11	1	0
Llama	0.96	0.03	0.97	0.02	1	0	1	0	1	0	0.42	0.57	0.96	0.03	0.83	0.16	0.96	0.03	0.64	0.35	0.97	0.02	0.46	0.53
Alpaca	0-67	0.32	0.73	0.26	1	0	0.97	0.023	0.97	0.02	0.54	0.45	0.66	0.33	0.76	0.23	0.64	0.35	0.78	0.21	0.66	0.33	0.52	0.47
Total	0.87 (0.14)	0.12 (0.14)	0.87 (0.12)	0.12 (0.12)	0.97 (0.04)	0.02 (0.04)	0.99 (0.01)	0.005 (0.01)	0.99 (0.01)	0.005 (0.01)	0.62 (0.25)	0.37 (0.25)	0.83 0.17)	0.16 (0.17)	0.90 (0.11)	0.09 (0.11)	0.90 (0.17)	0.09 (0.17)	0.85 (0.17)	0.14 (0.17)	0.84 (0.16)	0.15 (0.16)	0.62 (0.24)	0.37 (0.24)

Supplementary Table 9. Results of haplotype association analysis of synonymous *MC1R* gene for different taxa and traits.

Specie or subspecie	N	Hap	A82G	C92T	G112A	G259A	A265G	G376A	T383C	C901T	[1]GSS	[2]Hap-Score	[3]p-val	control.hf	[4]case.hf	[5]OR
Wild species	167	1	G	C	G	G	A	G	T	C	1.0618e-47	5.1001	3.3e-07	7.8e-02	0.2754	1.448
		2	A	C	G	G	A	G	T	C		6.2073	5.3e-10	1.7e-01	0.4880	1
		3	G	C	G	G	G	G	T	C		6.8630	6.7e-12	1.1e-03	0.2335	7.3e+14
Domestic species	173	4	A	C	G	A	A	G	T	C	1.0618e-47	33.546	7.9e-04	NA	5.1e-02	3.6e+17
		5	G	C	G	G	A	G	T	T		37549	1.7e-04	NA	6.4e-02	7.8e+16
		6	A	C	G	G	A	G	C	C		42865	1.8e-05	NA	6.7e-02	4.7e+17
		7	A	C	G	A	A	A	T	C		9.5761	1.0e-21	NA	3.4e-01	1.6e+29
Guanaco	82	2	A	C	G	G	A	G	T	C	1.5187e-49	15.596	7.6e-55	1.1e-01	0.9939	1
Northern guanaco	19	2	A	C	G	G	A	G	T	C	4.5256e-05	4.7568	1.9e-06	0.42229	1	1
Southern guanaco	63	2	A	C	G	G	A	G	T	C	1.246e-22	10.172	2.6e-24	0.18269	0.9920	1
Vicuña	85	1	G	C	G	G	A	G	T	C	5.6999e-61	11.010	3.3e-28	4.4e-02	0.5411	2.8e+14
		3	G	C	G	G	G	G	T	C		11.771	5.4e-32	NA	0.4588	5.6e+26
Northern vicuña	53	1	G	C	G	G	A	G	T	C	9.8058e-32	12.053	1.8e-33	NA	0.8679	1.8e+15
		3	G	C	G	G	G	G	T	C		-2.1875	2.8e-02	0.28070	0.1320	6.6e+04
Southern vicuña	32	3	G	C	G	G	G	G	T	C	4.7893e-30	11.799	3.9e-32	0.05185	1	7.0e+05
Llama	89	7	A	C	G	A	A	A	T	C	4.7004e-22	3.5499	3.8e-04	1.2e-01	2.8e-01	2.163
		6	A	C	G	G	A	G	C	C		6.3783	1.7e-10	NA	1.0e-01	2.7e+14
Alpaca	84	4	A	C	G	A	A	G	T	C	1.2514e-26	2.5080	1.2e-02	1.4e-02	0.0551	5.722
		5	G	C	G	G	A	G	T	T		6.9846	2.8e-12	NA	0.1345	1.9e+18
		7	A	C	G	A	A	A	T	C		7.6499	2.0118e-14	9.0e-02	0.4207	6.067
**Trait**	**N**	**Hap**	**A82G**	**C92T**	**G112A**	**G259A**	**A265G**	**G376A**	**T383C**	**C901T**	**[1]GSS**	**[2]Hap-Score**	**[3]p-val**	**control.hf**	**[4]case.hf**	**[5]OR**
Wild*	172	1	G	C	G	G	A	G	T	G	2.3659e-42	4.8287	1.3e-06	7.9e-02	0.2674	1.042
		2	A	C	G	G	A	G	T	A		5.6997	1.1e-08	1.8e-01	0.4738	1
		3	G	C	G	G	G	G	T	G		6.6592	2.7e-11	2.0e-03	0.2267	6.451
Black	14	7	A	C	G	A	A	A	T	C	0.0035773	3.3831	0.00071	0.16300	4.6e-01	3.328
Dark_brown	16	7	A	C	G	A	A	A	T	C	0.0072609	2.4234	0.01537	1.6e-01	3.7e-01	2.749
Light_brown	28	4	A	C	G	A	A	G	T	C	1.1574e-07	3.1752	0.00149	1.8e-02	1.0e-01	3.777
		6	A	C	G	G	A	G	C	C		3.2439	0.00117	1.7e-02	1.0e-01	5.274
White	37	7	A	C	G	A	A	A	T	C	1.7183e-09	6.4832	8.9e-11	1.2e-01	5.0e-01	4.144
		2	A	C	G	G	A	G	T	C		-2.846	4.4e-03	3.4e-01	1.4e-01	1

[1]Global Score Statistics p-val: The overall association between haplotypes and the response.
[2]The score for the haplotype, which is the statistical measurement of association of each specific haplotype with the trait.
[3]The asymptotic chi-square (1 df) P-value, calculated from the square of the score statistic.
[4]Estimated frequency of each haplotype in the population whit the trait.
[5]Odds Ratio based on haplo.glm model estimated coefficient for the haplotype.
Wild* corresponds to the layer colour present in wild animals that is also present in 4 llamas and 1 alpaca here studied.

Supplementary Table 10. Results of haplotype association analysis of synonymous *ASIP* gene for different taxa and traits.

Trait	N	Hap	C292T	G353A	del 57bp	[1]Gss	[2]Hap-Score	[3]p-val	control.hf	[4]case.hf	[5]OR
Wild	165	1	C	G	+	1.7e-37	13.313	1.9e-40	4.0e-01	1	1
Domestic	173	2	C	A	+	1.7e-37	5.433	5.5e-08	NA	1.3e-01	1.6e+82
		3	T	G	+		6.836	8.1e-12	NA	1.7e-01	1.9e+94
		4	C	G	-		8.028	9.8e-16	NA	2.6e-01	7.5e+84
Guanaco	81	1	C	G	+	6.3e-12	7.622	2.4e-14	6.0e-01	1	1
Vicuña	84	1	C	G	+	1.5e-12	7.809	5.7e-15	5.9e-01	1	1
Llama	89	3	T	G	+	1.4e-16	3.468	5.2e-04	0.0448	0.1481	3.5589
		4	C	G	-		8.008	1.1e-15	0.0489	0.3539	5.0594
Alpaca	84	3	T	G	+	4.5e-26	4.527	5.9e-06	0.0465	1.9e-01	4.8669
		2	C	A	+		8.816	1.1e-18	0.0062	2.5e-01	27.165
**Trait**	**N**	**Hap**	**C292T**	**G353A**	**del 57bp**	**[1]Gss**	**[2]Hap-Score**	**[3]p-val**	**control.hf**	**[4]case.hf**	**[5]OR**
Wild*	170	1	C	G	+	2.8e-32	12.351	4.8e-35	4.1e-01	0.9705	1
Dark_brown	16	4	C	G	-	0.0383	2.847	0.0044	0.1231	3.4e-01	2.3302
Light_brown	28	4	C	G	-	4.5e-06	2.117	3.4e-02	0.1215	2.5e-01	2.3445
		3	T	G	+		4.723	2.3e-06	0.0620	2.8e-01	4.6228
Crema	11	3	T	G	+	0.0012	3.374	7.3e-04	0.0692	3.1e-01	9.2863
		4	C	G	-		2.565	1.0e-02	0.1233	3.6e-01	5.8008
White	38	2	C	A	+	1.6e-05	2.405	1.6e-02	4.9e-02	1.4e-01	3.2143
		3	T	G	+		2.958	3.0e-03	6.5e-02	1.8e-01	3.0915
		4	C	G	-		2.221	2.6e-02	1.2e-01	2.3e-01	2.0901

[1]Global Score Statistics p-val: The overall association between haplotypes and the response.
[2]The score for the haplotype, which is the statistical measurement of association of each specific haplotype with the trait.
[3]The asymptotic chi-square (1 df) P-value, calculated from the square of the score statistic.
[4]Estimated frequency of each haplotype in the population whit the trait.
[5]Odds Ratio based on haplo.glm model estimated coefficient for the haplotype.
Wild* corresponds to the layer colour present in wild animals that is also present in 4 llamas and 1 alpaca here studied.

Supplementary Figure 1: Structural organization of *MC1R*. Details of substitutions labelled here are provided in Table 1.

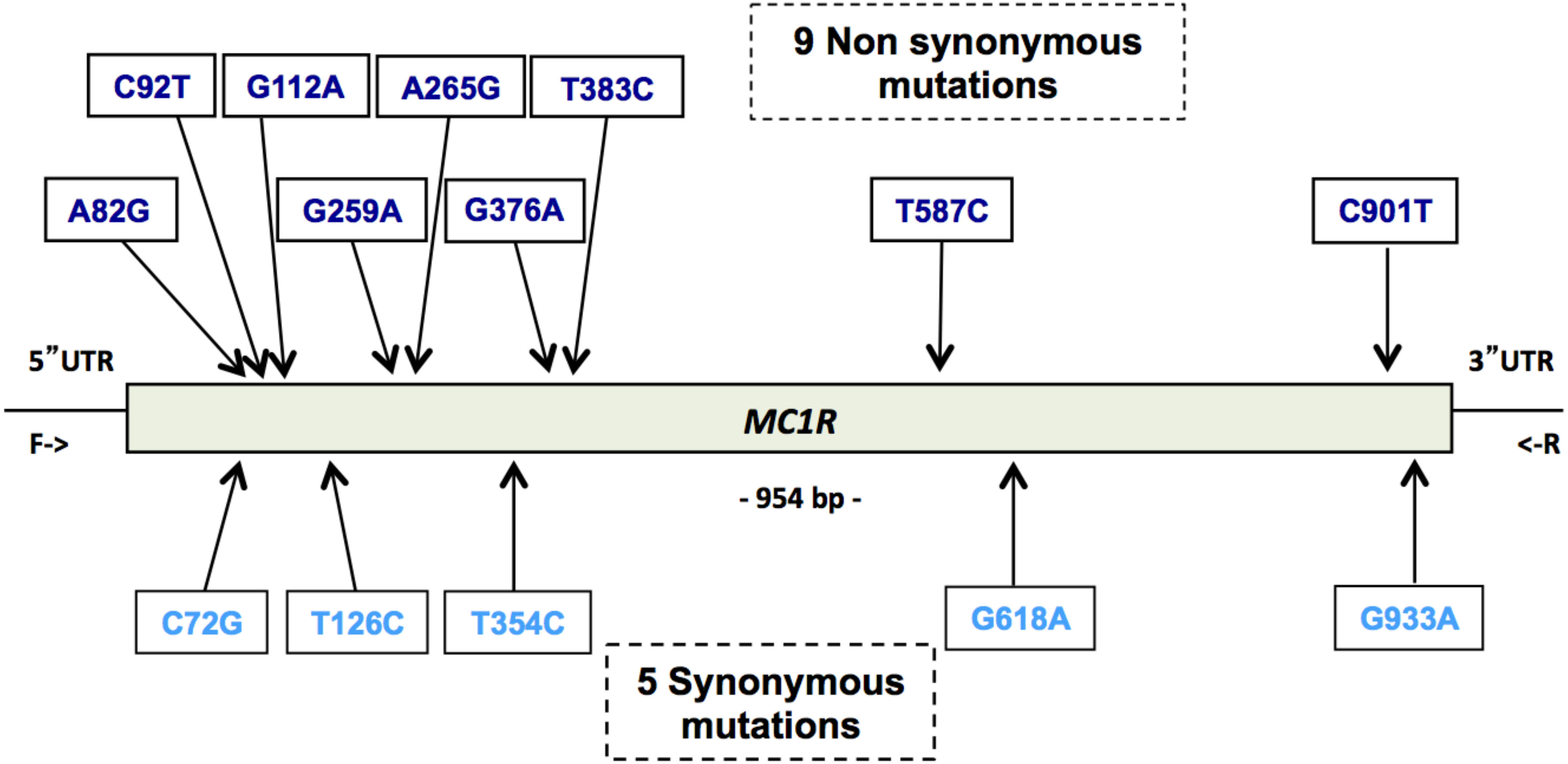

Supplementary Figure 2: Structural organization of *ASIP*. Additional details of substitutions are provided in Table 2.

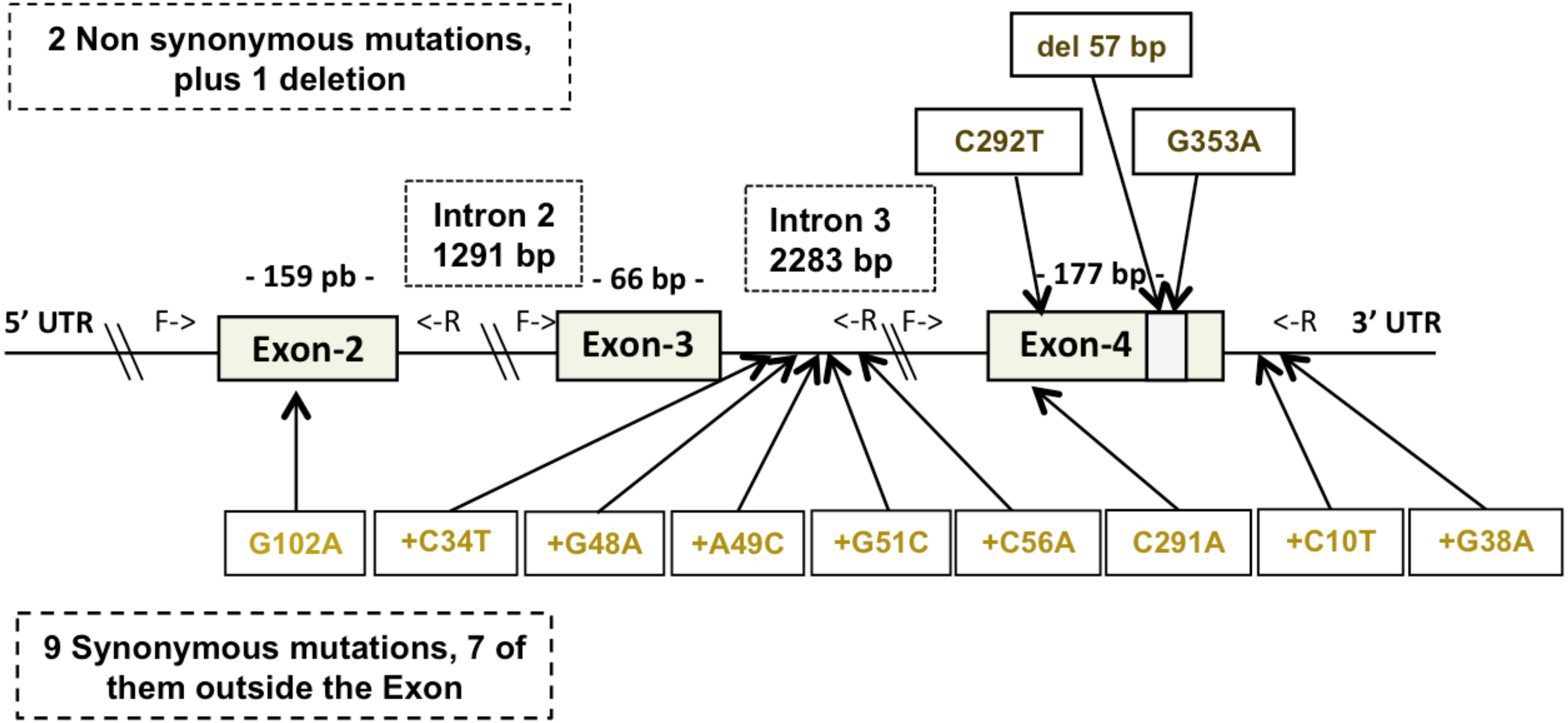

Supplementary Figure 3: Analyses were based on either the F_{ST}-outlier method in *MC1R* (**A**) and *ASIP* (**B**), implemented in the FLK statistic.

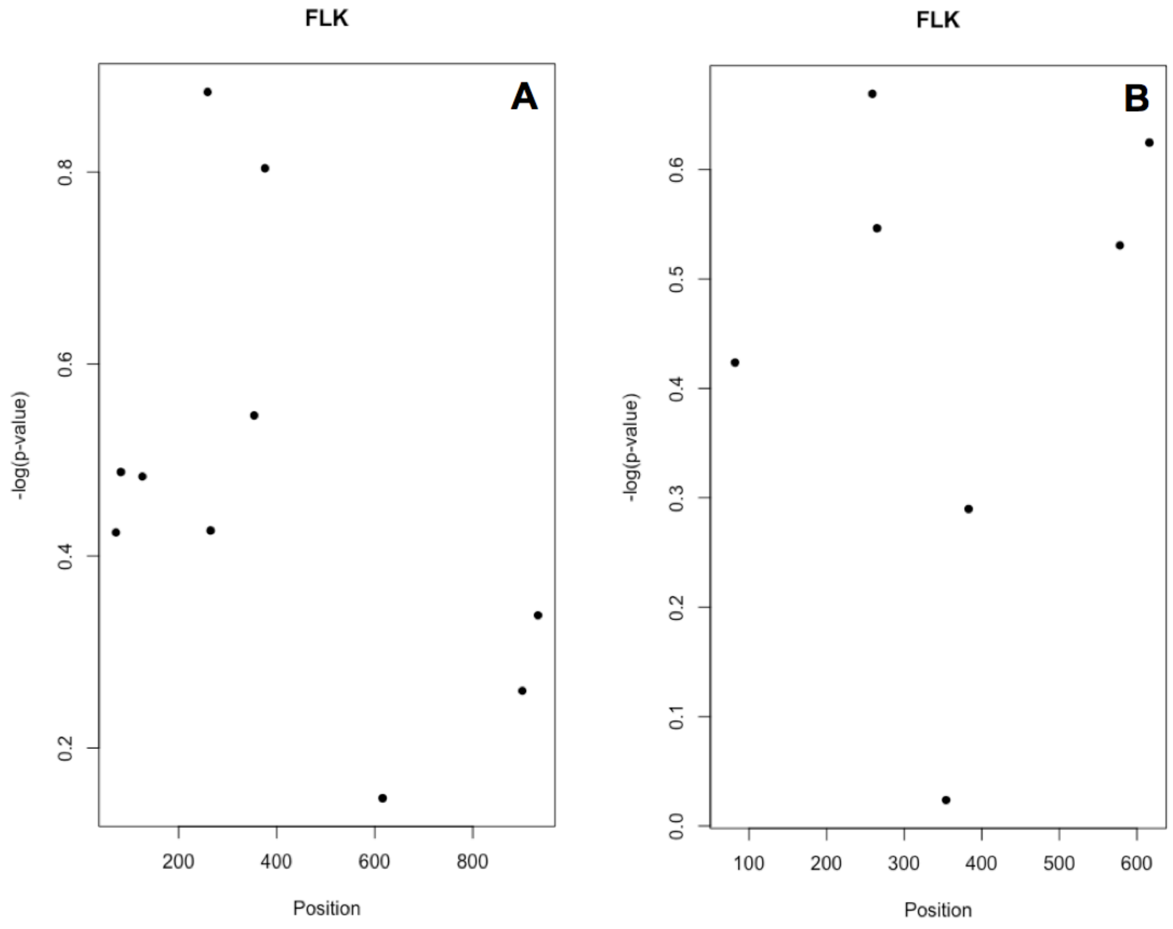

Supplement: Supplementary file 1 [file Data_Sheet_1.PDF]
